# Autofluorescence of Red Blood Cells Infected with *P. falciparum* as a Preliminary Analysis of Spectral Sweeps to Predict Infection

**DOI:** 10.3390/bios15020123

**Published:** 2025-02-19

**Authors:** Miguel A. Garrido-Tamayo, Alejandro Rincón Santamaría, Fredy E. Hoyos, Tamara González Vega, David Laroze

**Affiliations:** 1 Escuela de Física, Universidad Nacional de Colombia Sede Medellín, A.A: 3840, Medellín 050034, Colombia; 2 Vicerrectoría de Investigación y Postgrado, Universidad de La Serena, La Serena 1700000, Chile; 3 Grupo de Investigación en Microbiología y Biotecnología Agroindustrial—GIMIBAG, Universidad Católica de Manizales, Carrera 23 No. 60-63, Manizales 170002, Colombia; arincons@ucm.edu.co; 4 Grupo de Investigación en Desarrollos Tecnológicos y Ambientales—GIDTA, Universidad Católica de Manizales, Carrera 23 No. 60-63, Manizales 170002, Colombia; 5 Departamento de Energía Eléctrica y Automática, Facultad de Minas, Universidad Nacional de Colombia Sede Medellín, Carrera 80 No. 65-223, Robledo, Medellín 050041, Colombia; fehoyosve@unal.edu.co; 6 Tecnología Médica, Escuela de Ciencias de la Salud, Universidad Viña del Mar, Viña del Mar 2572007, Chile; tamara.gonzalez@uvm.cl; 7 Instituto de Alta Investigación, Universidad de Tarapacá, Casilla 7D, Arica 1000000, Chile; dlarozen@academicos.uta.cl

**Keywords:** fluorescence, intrinsic, RBC, malaria, EEM, excitation emission matrix

## Abstract

Malaria, an infectious disease caused by parasites of the genus *Plasmodium*—including the most lethal species, *Plasmodium falciparum*—alters the physicochemical properties of host red blood cells, including their intrinsic autofluorescence after infecting them. This exploratory study aims to investigate the possibility of using autofluorescence as a method for detecting infection in red blood cells. The autofluorescence spectra of uninfected and in vitro infected red blood cells with *Plasmodium falciparum* were monitored and compared across an excitation wavelength range of 255 to 630 nm. Principal Component Analysis revealed that only two wavelengths (315 and 320 nm), previously undocumented, were able to accurately differentiate infected from uninfected red blood cells, showing an increase in autofluorescence in the ultraviolet and blue regions. This phenomenon is hypothetically associated with the presence of natural fluorophores such as tryptophan, FAD, NADH, porphyrins, and lipopigments. To classify the samples, Linear Discriminant Analysis (LDA) was employed, and Wilks’ Lambda test confirmed that the discriminant function was significant, enabling correct classification of samples in more than 91% of cases. Overall, our results support the potential use of autofluorescence as an effective approach for detecting malaria parasite infection in red blood cells, with the possibility of implementation in portable devices for rapid field diagnostics.

## 1. Introduction

Malaria is caused by five species of the parasite genus *Plasmodium*, four of which (*P. falciparum*, *P. vivax*, *P. malariae* and *P. ovale*) infect humans, while *P. knowlesi* can be transmitted from monkeys to humans via Anopheles mosquitoes [1]. In 2022, the WHO reported 608,000 malaria-related deaths, with 96% concentrated in 29 countries; Nigeria and the Democratic Republic of the Congo accounted for 43% of the total [2].

Limited access to rapid, cost-effective therapies and diagnostics remains a challenge in low- and middle-income countries [3]. Although microscopy is the gold standard, its accuracy depends on the microscopist. Rapid diagnostic tests (RDTs), which are practical in remote areas, have limitations due to cost and quality control issues, while molecular methods, although sensitive, are expensive and require trained personnel [4,5,6,7,8,9,10]. Studies show that RDTs have an average sensitivity of 56.1% versus molecular methods such as PCR, while microscopy has a sensitivity of 89.2% for *P. falciparum* but only 8.3% for mixed infections with *P. vivax* [11,12]. A study by Opoku Afriyie et al. (2023), which included 1040 Ghanaian patients infected with *P. falciparum*, evaluated the performance of microscopy and RDTs in comparison to high-sensitivity var ATS quantitative PCR (var ATS qPCR). It was concluded that both tests failed to detect more than 40% of infections identified via qPCR var ATS [13].

Histidine-rich protein 2 (HRP2)-based RDTs for *P. falciparum* can produce false-negative results in cases where the parasites does not express this protein or its homolog, HRP3 [2].

Molecular techniques such as LAMP, microarrays, and mass spectrometry are useful in research but are uncommon in the field because of their high costs [14]. Microfluidic tests have been implemented to concentrate infected red blood cells, allowing more sensitive detection and improving the diagnostic accuracy of tests such as PCR [15,16,17]. In addition, automated cell counters have been implemented and developed for malaria diagnosis using flow cytometry [18]. More recently, the Sysmex XN-31 has been tested for malaria diagnosis in Colombia [19] and Kenya [20] and asymptomatic blood donors in Malawi, successfully detecting gametocytes [20]. Similarly, flow cytometry has been used to detect autofluorescence in erythrocytes with anemia and to identify parasites inside erythrocytes or liver cells using fluorescent markers [21,22,23,24,25]. Moreover, spectroscopic methods such as Raman spectroscopy (RS), near-infrared spectroscopy (NIRS), and mid-infrared spectroscopy (MIRS), combined with autonomous learning techniques, have also been employed to detect malaria [26,27,28].

Blood autofluorescence is a topic of great interest at present, as it has potential applications in the detection and diagnosis of different diseases without fluorescent markers. However, it has certain limitations, such as a lack of specificity due to the scarcity of natural fluorophores in cells. Endogenous fluorophores include hemoglobin porphyrin, aromatic amino acids (tryptophan, tyrosine, and phenylalanine), and coenzymes involved in metabolic and functional processes of the cell such as flavin (FAD), pyridine nucleotides (NADH and NADPH), and lipopigments [29].

Hemoglobin is the main component of red blood cells. Previous studies indicate that excitation by a single photon does not induce autofluorescence because the iron contained in the heme group, which is the main fluorophore in hemoglobin, acts as an electron acceptor. Consequently, two-photon excitation is needed for hemoglobin to fluoresce [30,31,32,33].

Light-emitting diode (LED) technology has been used to induce fluorescence in whole blood from healthy patients. Experiments showed a red shift in emission (627 nm) with increasing blood concentration when excited at 407 nm. Similarly, a shift to 607 nm was observed when using an excitation wavelength of 508 nm. Therefore, the shorter the wavelength of the excitation light (more energetic), the fluorescence peak shifts to longer wavelengths at the same concentration, with absorption being the main cause of this behavior [34].

In *Plasmodium falciparum* malaria, LEDs have also been used for multispectral imaging to distinguish infected from uninfected red blood cells and to differentiate between ring forms of trophozoites [35]. Absorption and scattering studies of RBCs infected with *P. falciparum* at different stages of the parasite have also been tested using UV–visible spectroscopy, developing theoretical models for the interpretation of the results [36]. According to Opoku-Ansah et al. (2016), when fluorescence was induced in whole blood with a 405 nm laser, the autofluorescence of blood from patients with *P. falciparum* malaria decreased as parasite density increased. This was due to the presence of hemozoin and reduced hemoglobin, which causes a red shift [37].

In a recent bibliometric study conducted by our team (Manuscript under review), 349 papers published over 44 years on diagnostic methods for *P. falciparum* malaria were analyzed. It was observed that microscopy, rapid diagnostic tests, and molecular tests remain the most researched topics in malaria diagnosis. Other diagnostic methods have not achieved significant advancements that would justify their adoption by the WHO as field diagnostic tools [38]. This presents a valuable opportunity for the development of innovative diagnostic techniques for malaria. Although previous studies have identified differences in the fluorescent properties of red blood cells infected with *Plasmodium falciparum*, specific wavelengths in the UV range that enable robust discrimination between infected and uninfected samples have yet to be identified. Moreover, the potential application of these properties in developing portable diagnostic tools remains largely unexplored.

This exploratory study aimed to assess the feasibility of using autofluorescence to detect erythrocytes infected with *Plasmodium falciparum*, the most lethal and prevalent malaria species in endemic regions, responsible for the majority of severe cases and deaths related to the disease.

To achieve this, uninfected (u-RBC) and infected (i-RBC) erythrocytes were excited across a wavelength range of 255 to 630 nm, producing excitation–emission matrices (EEMs) that served as spectral fingerprints of the blood samples. Principal Component Analysis (PCA) was employed to identify excitation wavelengths capable of distinguishing between the samples, pinpointing spectral regions with significant variations. The results demonstrate the potential of autofluorescence as an effective method for identifying erythrocyte infections caused by malaria parasites.

## 2. Materials and Methods

### 2.1. Sample Preparation

Samples of healthy and infected red blood cells were provided by the Malaria Group of the Universidad de Antioquia (UdeA), Medellín, Colombia. As shown in Table 1, a total of 12 red blood cell samples were processed: six from blood group A-positive donors without leukocytes and other six samples in vitro infected with *P. falciparum*, with an average parasitemia of 3.69%, as determined via Giemsa staining [39]. The samples were evaluated under an optical microscope using standard procedures [40].

In addition to the 12 samples originally described (6 uninfected RBCs, 5 infected with *P. falciparum* strain FCR3, and 1 FCB1), additional analyses of 6 samples infected with *P. falciparum* strain 3D7 and 6 additional normal RBC samples obtained under similar conditions were included (see Appendix A). In total, 24 samples were analyzed, allowing for the assessment of greater variability in autofluorescence characteristics.

Uninfected samples (u-RBC) were collected in EDTA tubes to prevent clotting and were processed immediately or stored at 4 °C for up to 24 h before analysis. Infected samples (i-RBC) were cultured in vitro with *Plasmodium falciparum* strains FCR3, FCB1, and 3D7 in RPMI 1640 medium (Sigma-Aldrich, Burlington, MA, USA) supplemented with group O human plasma. Samples were stored at 37 °C under controlled CO_2_ conditions until processing.

**Figure 1:**
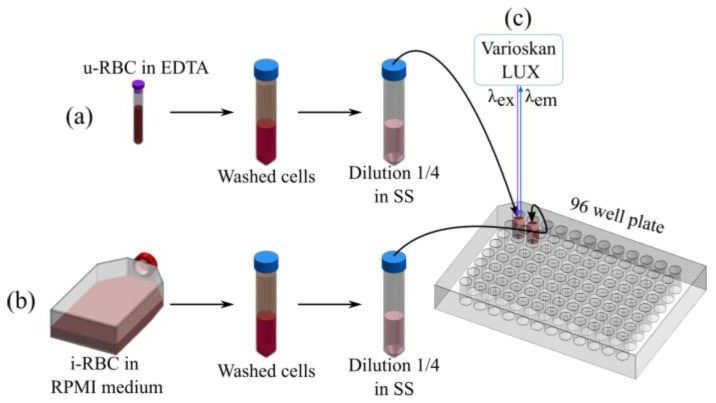
Schematic of the experimental process for washing healthy erythrocytes (u-RBC) anticoagulated with EDTA (**a**) and infected erythrocytes (i-RBC) from RPMI culture media (**b**), followed by dilution and reading. The washed cells were diluted in saline solution (SS) and spread in a 96-well plate, achieving a final hematocrit of 25%, before excitation (λ_ex_) and emission (λ_em_) readings were taken using the Varioskan LUX (**c**).

To wash the cells and remove the medium, 250 µL of u-RBCs were suspended in 1.5 mL of 0.9% NaCl saline solution (SS). Similarly, the i-RBCs were washed in the same manner. Samples were mixed via inversion and centrifuged at 2500 rpm for 5 min. This process was repeated 4 times until a clear supernatant was obtained. A final solution was prepared with 50 µL of concentrated RBCs in 150 µL of SS to obtain a hematocrit (HCT) of 25% in each well (Figure 1).

The results shown in this research correspond to the analysis of red blood cells infected with *P. falciparum* strains FCR3 and FCB1 since they share significant similarities [41].

### 2.2. Spectrofluorimetry

The autofluorescence of i-RBCs and u-RBCs was measured using the Variskan Lux (Thermo Scientific, manufactured in Waltham, MA, USA), a microplate reader with a Xenon flash lamp and double monochromator, one for sample excitation and the other for fluorescence emission. The excitation of the samples was carried out from the top, as was the acquisition of the fluorescence, as illustrated in Figure 1. The Varioskan LUX performs automatic calibration at the time of measurement every 7.5 min. This includes correction for fluctuations in lamp intensity and the use of reference sensors that adjust the results to ensure the accuracy and reproducibility of the measurements.

The EEMs of u-RBCs and i-RBCs were recorded in 200 µL of previously washed and diluted samples. The schematic configuration of the study included a sweep in the excitation (λ_ex_), running from 255 nm to 630 nm, with a bandwidth of 5 nm and steps of 5 nm, spanning a total of 76 excitation wavelengths. The fluorescence emission (λ_em_) of the samples was measured for each excitation wavelength, ranging from λ_ex_ +22 nm to 840 nm, with 2 nm increments. Before each measurement, the samples were mixed for 5 s at 600 rpm, and readings were taken from the top of the cuvette at a constant temperature of 25 °C, with an integration time of 100 ms for each reading.

To generate the matrix plot or plots of the EEMs, the result files were processed using Wolfram Mathematica 10.2 and MATLAB R2023b (The Mathworks, Natick, MA, USA). Negative values were set to zero, matrices were averaged, and in some cases, normalized to the maximum intensity obtained. Individual curves for each excitation wavelength were smoothed, and emission peaks were plotted using Gaussian curves in OriginPro 2018.

### 2.3. Statistical Treatment

A Principal Component Analysis (PCA) was conducted using Past 4.09 [42] on the EEM to identify the excitation wavelengths that could differentiate malaria-infected red blood cell samples from healthy red blood cells. This methodology is justified, as PCA has previously been employed on Raman spectra obtained from macrophages that had phagocytosed hemozoin from malaria parasites, allowing spectral changes to be observed compared to macrophages without hemozoin [43]. On the other hand, PCA has been used to differentiate corneal pathogenic microorganisms based on their autofluorescence [44]. Furthermore, Linear Discriminant Analysis (LDA) was performed to obtain the discriminant equation for classifying the samples. Additionally, Leave-One-Out Cross-Validation (LOOCV) was used to maximize the evaluation of each sample in the dataset. In each iteration, one sample was used for testing, while the remaining samples were used for training, repeating the process 24 times. For comparison, a k-fold validation was also performed with k = 5, generating average metrics and standard deviations [45]. These procedures were performed in Python 3.12.5 (Python Software Foundation, Wilmington, DE, USA) with Visual Studio Code 1.92.2 (Microsoft, Redmond, WA, USA).

## 3. Results

### 3.1. Signal Processing

**Figure 2:**
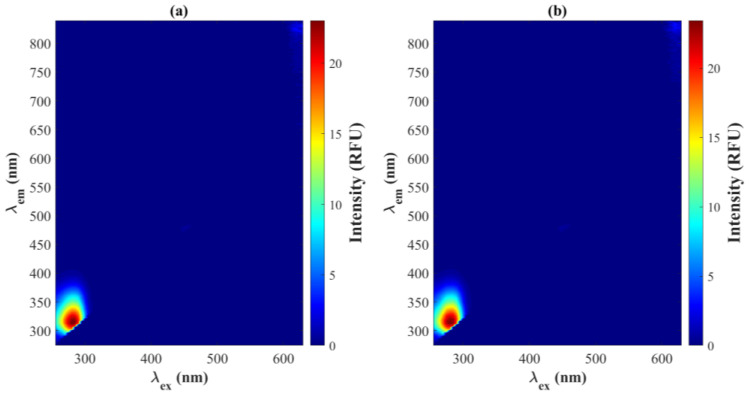
Excitation–emission matrices (EEM) for (**a**) u-RBCs and (**b**) i-RBCs. Key differences between the signals are not visible to the naked eye, which prompted the use of Principal Component Analysis (PCA) to identify significant variations in fluorescence.

To investigate whether excitation wavelengths could differentiate between i-RBCs and u-RBCs, we analyzed autofluorescence spectroscopy arrays for both cell types. By evaluating the averages of the EEMs, significant autofluorescence emission was observed in the spectral region between 278 and 390 nm when excitation wavelengths between 255 and 310 nm were used, with its maximum peak at 280 nm in both arrays (Figure 2a,b).

The difference in fluorescence between the i-RBC and u-RBC arrays was calculated for each excitation value, with the largest difference observed at 275 nm. However, this difference does not necessarily guarantee adequate differentiation between the samples. It is possible that more significant differences are present at wavelengths with lower intensity. Therefore, a multivariate statistical analysis was performed in order to extract relevant information from both matrices and detect subtle variations that could be key to differentiating the samples.

To reinforce the robustness of the method, six samples infected with the 3D7 strain of *Plasmodium falciparum* and their respective normal counterparts were included for statistical analysis. These additional results are presented in Appendix A since the main focus of the manuscript is on the preliminary evaluation of the FCR3 strain.

### 3.2. Principal Components Analysis (PCA)

**Figure 3:**
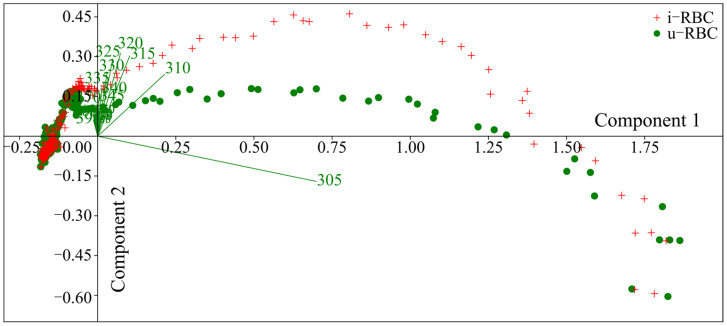
Biplot of the first two principal components of the PCA in the excitation segment from 305 nm to 400 nm, showing that the wavelengths from 305 to 335 nm (green lines) have higher loadings for differentiating i-RBCs (red crosses) from u-RBCs (green points).

Since the EEMs contain irrelevant information (values with zero intensity), a Principal Component Analysis (PCA) was conducted to reduce the dimensionality of the matrices. This analysis allowed us to identify the variables with the highest loadings in the covariance of the samples and to plot the new components. The PCA was performed in segments since the analysis of the complete matrices did not show many differences. The first segment covered excitation wavelengths from 255 to 300 nm; the second, from 305 to 400 nm; the third, from 405 to 500 nm; and the last, from 505 to 630 nm. Only the second segment, from 305 to 400 nm of excitation, showed significant differences between the u-RBC and i-RBC matrices. As shown in Figure 3, the biplot of the first two principal components in the second segment reveals that seven wavelengths, from 305 to 335 nm (green lines), have higher loadings for differentiating i-RBCs (red crosses) from u-RBCs (green dots).

In the PCA analysis (Table 2), principal component 1 (PC1) explained 91.69% of the total variance, while principal component 2 (PC2) explained 29.30%. The wavelengths that contributed most significantly to PC1 were 305 nm (coefficient 0.91694) and 310 nm (coefficient 0.30807). For PC2, the highest contributions were observed at wavelengths 320 nm (coefficient 0.42394), 325 nm (coefficient 0.39272), and 315 nm (coefficient 0.39064). These results reinforce that the 315 and 320 nm wavelengths are key in differentiating infected and uninfected samples.

**Figure 4:**
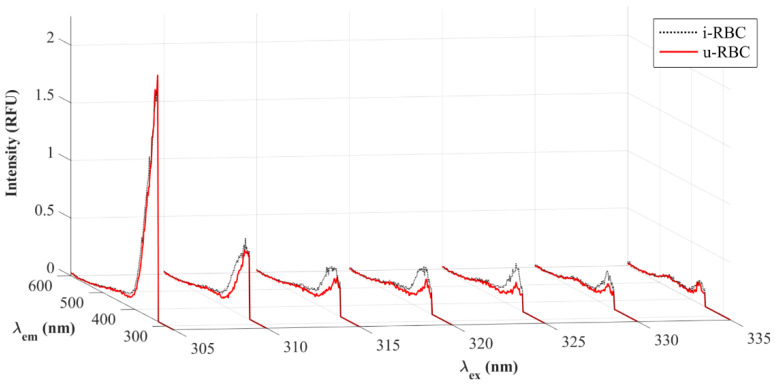
Fluorescence intensities of u-RBCs and i-RBCs. The spectra shown correspond to the seven excitation wavelengths obtained in the PCA.

Figure 4 shows the seven excitation wavelengths with the highest charges resulting from the PCA. Wavelengths of 305 and 335 nm do not show much differences in intensities between healthy and infected samples. In contrast, the others show an increase in fluorescence intensity for i-RBCs (black dotted lines) compared to u-RBCs (red lines).

**Figure 5:**
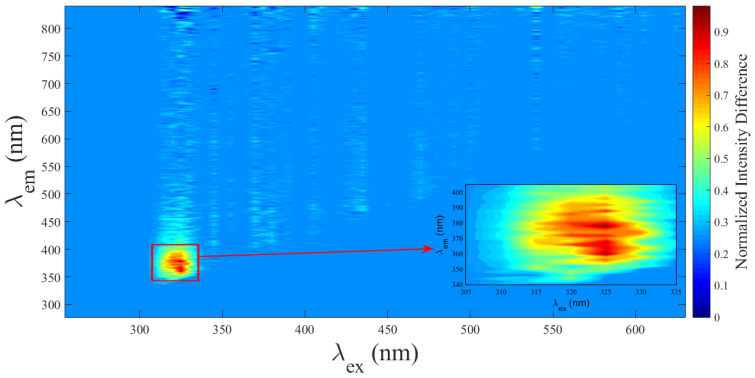
Normalized fluorescence difference in fluorescence intensity between i-RBC and u-RBC. The largest average difference occurs at excitation wavelengths between 315 and 330 nm (see the large colored spot in the zoomed-in graphic).

The mean difference in fluorescence intensity between infected (i-RBC) and uninfected (u-RBC) red blood cells, normalized to the maximum intensity of u-RBC, was calculated for each excitation wavelength using the mean value theorem. To fit the differences, each column of data (corresponding to an excitation wavelength) was assigned a weight based on the average of the differences observed in that column. This weight reflects the relative importance of each excitation. The calculated weights, which represent the magnitude of the normalized mean differences, are as follows: 0.0728 for 325 nm excitation, 0.0653 for 320 nm excitation, 0.0582 for 315 nm excitation, and 0.0545 for 330 nm excitation. These values show that the largest differences are observed in the excitation range from 315 to 330 nm. The normalized mean differences are shown in Figure 5. The zoomed-in box in the spectral region from 305 to 335 nm highlights the specific differences in fluorescent signals, which are less evident on the full-scale graph. It indicates that the largest average differences are observed in the excitation range from 315 to 330 nm. This confirms the PCA result.

If the mean difference is significant, it indicates that, on average, each element in the i-RBC matrix has a higher value than the corresponding value in the u-RBC matrix for that particular element. This suggests that the i-RBCs have undergone a specific process or condition that has systematically increased the spectral response of their autofluorescence compared to the u-RBCs.

**Figure 6:**
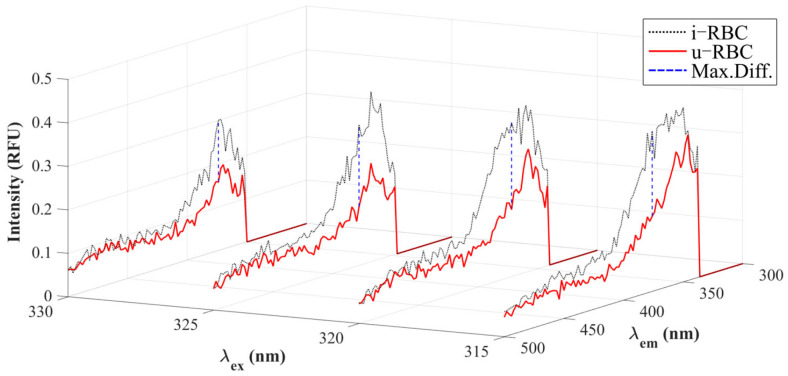
Maximum differences (Max. Diff.) in autofluorescence intensity between infected and uninfected samples corresponding to the excitation wavelengths from 315 to 330 nm, selected as those with the highest average normalized difference.

Next, we analyzed the four wavelengths that presented the maximum normalized mean differences. Figure 6 shows the maximum differences between the samples for the selected wavelengths (dashed blue lines), with the highest values being 0.2002 for 320 nm excitation, 0.1955 for 315 nm, 0.1890 for 325 nm, and 0.1423 for 330 nm.

Only the two excitation wavelengths with the largest differences (315 and 320 nm) were selected for subsequent spectral analyses. Each spectrum was smoothed using the Savitzky–Golay method, with a 17-point window and polynomial order 3. To identify peaks within the smoothed signal, a manual residual peak fit was used, identifying the maximum peaks and fitting a sum of individual Gaussian functions that best represented the smoothed signal.

Total fluorescence as a function of emission wavelength is shown in Equation (1). The type of peak used is the Gaussian amplitude fit model.

(1) $$F(\lambda )=\sum_{i=1}^{n}A_{i}\times e^{-2{\left(\frac{\lambda -\lambda_{ci}}{w_{i}}\right)}^{2}},$$

where

$A_{i}$ is the fluorescence amplitude of each peak *i*;
$\lambda_{ci}$ is central wavelength of the Gaussian peak *i*;
$w_{i}$ is the full width at half maximum of each Gaussian bell (FWHM);
n is the total number of Gaussian peaks being summed.

**Figure 7:**
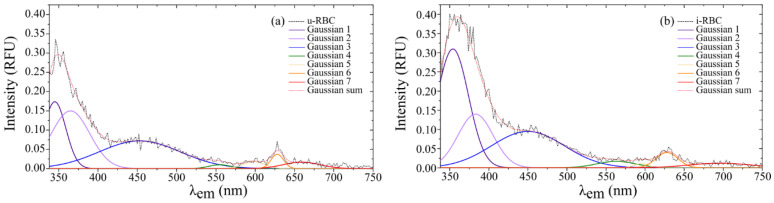
Fluorescence intensities of (**a**) healthy (u-RBC) and (**b**) infected (i-RBC) samples when excited at 315 nm and their corresponding Gaussian peak sums (dotted red line) that best fit the smoothed signal.

Equation (1) can be separated to represent each of the fluorophores or group of fluorophores in the sample responsible for each emission peak. Figure 7a,b shows the fluorescence peaks of the u-RBC and i-RBC samples, respectively, when excited with 315 nm and their corresponding Gaussian peak sums, consisting of a total of seven peaks.

**Figure 8:**
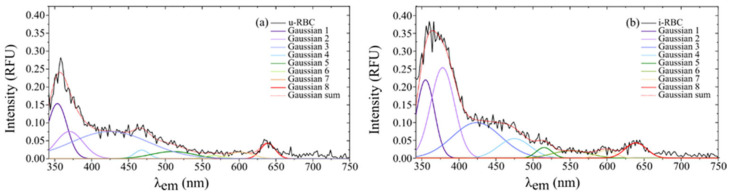
Fluorescence intensities of (**a**) healthy (u-RBC) and (**b**) infected (i-RBC) samples when excited at 320 nm and their corresponding Gaussian peak sums (dotted red line) that best fit the smoothed signal.

Similarly, Figure 8a,b shows the fluorescence peaks of the u-RBC and i-RBC samples, respectively, when excited at 320 nm and their corresponding Gaussian peak sums, which consist of a total of eight peaks.

**Figure 9:**
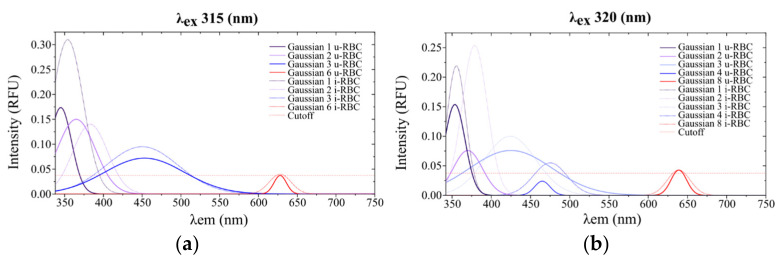
Gaussian peaks for u-RBC and i-RBC exceeding the 0.0375 RFU cutoff point for excitation wavelengths (**a**) 315 nm and (**b**) 320 nm.

In Figure 9, the horizontal dashed red line is set as the cutoff intensity for the peaks, with a value of 0.0375 RFU. Signals below this threshold do not exhibit clear peaks in the original data and are likely related to background noise. The figure shows the fitting curves of autofluorescence Gaussian peaks exceeding the 0.0375 RFU cutoff line, corresponding to uninfected (u-RBC) and infected (i-RBC) red blood cells under two different excitation wavelengths: 315 nm and 320 nm. In both plots, i-RBCs show Gaussian peaks with higher intensities compared to u-RBCs. This is particularly evident in the ultraviolet (UV), violet, blue, and green (350 to 500 nm). Additionally, a red shift in the autofluorescence peaks for i-RBCs (dotted lines) is observed in the regions from 338 to 378 nm under both excitation wavelengths. In contrast, in the red region (around 628 to 639 nm), the variation between samples is minimal. At 315 nm excitation, only four peaks stand out in both samples, while at 320 nm excitation, four peaks are prominent in the healthy sample and five in the infected sample. Furthermore, at the 320 nm excitation, the third peak shows a violet shift, and a new peak appears at around 475 nm, where the intensity in the i-RBCs exceeds 0.0375 RFU.

When comparing both excitation wavelengths, the peaks and their distribution change. For 315 nm excitation, significant peaks are observed in the UV, violet, and blue regions, with an additional peak in the red region. For 320 nm excitation, peaks also manifest in the UV and blue regions, but with a different distribution and more pronounced peak appearance in the violet and red regions. This indicates that the excitation wavelength influences which fluorophores are excited and thus which emissions are detected. Table 3 shows the characteristics of the different peaks obtained from the Gaussian curve fitting of the fluorescence signals for both 315 nm and 320 nm excitation wavelengths. A maximum mean intensity was obtained for u-RBCs at 345 nm with 315 nm excitation, with a 9 nm right shift in i-RBCs and a 78% increase in fluorescence in infected samples. For excitation at 320 nm, a maximum mean intensity was obtained for u-RBCs at 353.6 nm, with 1.4 nm redshift in i-RBCs and a 43% increase in fluorescence. A remarkable change appears in the second and fourth Gaussian peaks, with an increase of 234% (from 0.0760 RFU in u-RBC to 0.2540 RFU in i-RBC) and 129% (from 0.024 RFU in u-RBC to 0.055 RFU in i-RBC) in i-RBC with respect to u-RBC.

The Gaussian peaks in the red region of the spectrum (peak 6 at 315 nm and 8 at 320 nm) have little variation in amplitude and shift between u-RBC and i-RBC, which may suggest that the components responsible for the fluorescence in this region are less susceptible to changes induced by *Plasmodium falciparum*. This peak is related to the porphyrin present in both hemoglobin and hemozoin produced by the parasite [46].

**Figure 10:**
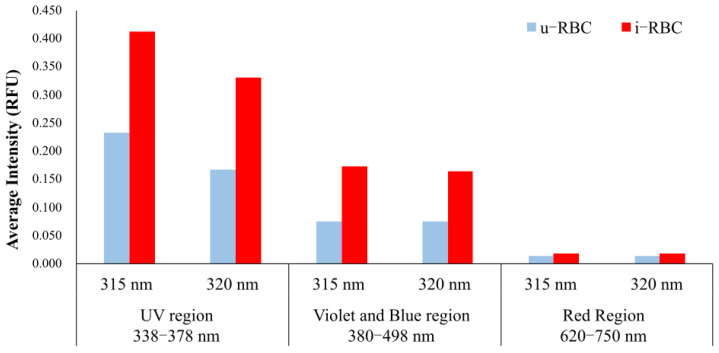
Mean intensity in the ultraviolet, violet-blue, and red regions for the two excitations (315 nm and 320 nm) in u-RBC and i-RBC infected with *P. falciparum*.

The average intensity in the UV (338–378 nm), where the Gaussian peaks 1 and 2 are located, as well as in the violet-blue (380–498 nm) and red (620–750 nm) regions of the two excitations (315 nm and 320 nm) for u-RBCs and i-RBCs, is shown in Figure 10. The autofluorescence of i-RBCs increased in the violet region by 77% and 97% when excited with 315 nm and 320 nm, respectively. In addition, the bar plot of the average intensity in the violet-blue region showed an increase of 129% with excitation at 315 nm and 119% with excitation at 320 nm. In the red region of the spectrum, the intensities remained relatively constant for both excitations. In summary, the results demonstrate an increase in autofluorescence in the parasitized samples after excitation with 315 and 320 nm.

Additional results indicated that the excitation wavelengths of 315 and 320 nm were unable to differentiate parasitemia in the samples, possibly due to the low intensity of the radiation emitted by the xenon lamp. This finding highlights the need for more powerful excitation sources, such as LEDs or lasers, to improve the sensitivity of the method.

### 3.3. Linear Discriminant Analysis (LDA)

To confirm the differentiation between healthy samples and those infected with *P. falciparum*, a Linear Discriminant Analysis (LDA) was performed using the maximum emission results obtained in the UV and violet-blue regions with excitations at 315 nm and 320 nm. The analysis yielded the following equation with the unstandardized coefficients:

(2) D=5.3268−1.1583×UV intensity−41.0590×Violet_Blue intensity.

Equation (2) calculates the discriminant value (D) for each sample on the maximum intensities from the two regions. This discriminant value places the sample as either healthy or infected, depending on whether it is below or above 0.

The confusion matrix is presented in Table 4. Out of the 12 healthy samples, all were correctly classified. Among the infected samples, two were misclassified, resulting in 22 out of 24 samples being accurately identified. This corresponds to an accuracy of 91.67%, suggesting that the method could be effective for detecting samples infected with *P. falciparum*.

It should be noted that the analysis included a total of 24 samples (12 infected and 12 non-infected). Although the misclassification of two infected samples partially limits the accuracy, the overall results highlight the potential of the method as a diagnostic tool, subject to future improvements.

To determine whether the discriminant function is significant, the Wilks’ Lambda test was used [47,48]. This test evaluates whether the means of the groups differ significantly as a function of the predictor variables. The results of the statistical significance analysis for the discriminant function are as follows: Wilks’ Lambda = 0.5208, suggesting that there is approximately 52% discrimination between the classes (“Healthy” and “Infected”). Lower values indicate better discrimination between classes, so this value already suggests good discriminative ability. The F statistic = 10.1213 indicates that this separation is significant. This F value is used to determine the significance of differences between group means. The associated *p*-value is 0.0008, which is significantly lower than 0.05, indicating that the discriminant function is statistically significant at the 95% confidence level. This clearly demonstrates that the variables used (mean intensities in the UV and blue-violet spectral bands) capture the real differences between healthy and infected samples, despite the moderate discrimination obtained from the Wilks’ lambda value. Leave-One-Out Cross-Validation (LOOCV) showed an average accuracy of 91.7%, while the k-fold Cross-Validation approach (k = 5) performed 92% ± 9.8%. These cross-validation results consolidate the robustness of the model, while the coefficient analysis highlights the importance of violet-blue as a key marker for classification. This suggests that the model could be a reliable and practical tool for detecting malaria infections based on the intrinsic autofluorescence of red blood cells.

## 4. Discussion

In this study, using a xenon lamp as the excitation source, we found two wavelengths in the UV region (315 and 320 nm) that allow differentiation of *Plasmodium falciparum* malaria-infected red blood cells (i-RBCs) from uninfected red blood cells (u-RBCs) due to increased autofluorescence in the UV, violet, and blue regions. The use of a xenon lamp as the excitation source was an appropriate choice for these preliminary studies, as it allowed us to determine optimal wavelengths for differentiating the samples. We recognize that laser-based systems could improve the sensitivity of the method, but their high cost and complexity limit their feasibility for portable applications. The goal of this work is to move towards the development of an inexpensive and portable device using high-intensity LEDs as the excitation source, which would facilitate its implementation in clinical settings and regions with limited resources.

While the results of this study show significant differentiation in autofluorescence between infected and uninfected erythrocytes, we recognize that the sample size (12 uninfected and 12 infected samples) may limit the generalizability of these findings. This relatively small sample size may not fully capture the variability in autofluorescence associated with individual factors, such as genetic and environmental differences or varying levels of parasitemia. Therefore, the results should be interpreted as preliminary and require validation in studies with a larger sample size. The use of LOOCV and k-Fold Cross-Validation was critical in this study because of the small sample size. These techniques maximize the utilization of available data to evaluate model efficacy, which is a key consideration in preliminary research with limited datasets. Although LOOCV ensures that each sample is evaluated individually, which is particularly useful for capturing variation in small samples, k-Fold offers a balance between stability and computational efficiency [45]. The consistent results obtained with both methods validate the robustness of the developed model, highlighting its potential as a reliable and practical tool for detecting malaria infections through the intrinsic autofluorescence of red blood cells. This provides a solid foundation for future research involving larger and more diverse sample sizes to further validate its applicability and generalizability.

Our results can be explained by the fact that i-RBCs undergo structural and functional alterations due to the metabolic processes of *P. falciparum*, the production of hemozoin, and the alterations induced by the maturation phase of the parasite, which lead to the accumulation of new fluorescent compounds [49]. The peaks observed in the ultraviolet, violet, blue, and red regions could be related to fluorophores such as tryptophan, flavin adenine dinucleotide (FAD), nicotinamide adenine dinucleotide (NADH), and heme porphyrins [29]. We recognize that the autofluorescence signal is influenced by these natural fluorophores, the concentration of which may vary between individuals and under different physiological conditions. These variations could lead to inconsistencies in practical applications. In this in vitro study, such variations were minimized using homogeneous RBC samples and standardized cultures of *P. falciparum*. However, future research should consider evaluating more diverse populations, implementing signal normalization, and using advanced statistical analysis to address this limitation.

The malaria parasite lyses red blood cells to continue its asexual reproductive cycle, and all fluorophores, which are products of red blood cell disintegration, are released into the plasma. In a study by Masilamani et al., 2014, plasma obtained from EDTA-anticoagulated whole blood from patients with and without malaria was analyzed. They found that the synchronous excitation spectra showed three significant bands in normal and infected patients: one at 280 nm due to tryptophan, another at 380 nm due to the coenzyme NADH, and the other at 460 nm due to FAD. Although all three bands appeared in both samples, the relationship between the peaks manages to differentiate the malaria samples. In the fluorescent emission spectrum of the two samples, they also observed three peaks when excited at a wavelength of 400 nm: one emission band at 460 nm due to FAD, another at 585 nm due to the basic form of porphyrin, a protein found in hemoglobin, and the other at 635 nm due to the neutral form of porphyrin [50]. The differences in fluorescence emission wavelengths observed in the basic and neutral forms of porphyrin are due to variations in their protonation state, which, in turn, affect the electronic structure and autofluorescence properties of the molecule. The hemoglobin-specific porphyrin is protoporphyrin IX, which is part of the heme group, and the fluorescence emission spectrum in the condensed crystalline phase of iron(III) protoporphyrin IX isolated from *P. falciparum* cultures as a malaria pigment (hemezoin) shows a maximum emission around 626 nm (red region of the spectrum) when excited at 405 nm. However, its contribution to the autofluorescence of anhydrous hematin and hemezoin crystals is limited compared to the intrinsic fluorescence of the condensed heme phase, which is around 577 nm [51]. In our case, the intensity of the peaks in the red region of the spectrum (620 a 675 nm) does not vary significantly between u-RBCs and i-RBCs. This suggests that the components responsible for the emission in this region are not significantly affected by infection or are less sensitive to it, indicating a relative stability of certain cellular components. Nevertheless, a slight broadening of the peak is observed in the infected samples for the two excitations (315 and 320 nm); a higher FWHM may indicate that the energy transitions responsible for the emission are more distributed, reflecting a greater dispersion in the levels of molecular energies. The breakdown of hemoglobin, which increases fluorescent products in plasma, such as heme, is relevant to the findings of our study on the importance of hemoglobin metabolites in autofluorescence and their alterations by the parasite.

Previous studies conducted by Opoku-Ansah et al., 2016, have shown differences in autofluorescence of malaria-infected cells, where autofluorescence decreases with increasing parasite density in infected blood and reaches a maximum emission at 612 ± 1 nm. This inconsistency with our results may be supported by the fact that different excitation sources and wavelengths were used, such as a 405 nm laser. Additionally, we used washed red blood cells suspended in saline solution instead of whole blood with ethylenediaminetetraacetic acid (EDTA) to quantify autofluorescence. Both studies agree that there is a red shift in parasitized samples [37]. However, unlike the studies of Masilamani and Opoku-Ansah, our use of a xenon lamp showed no significant difference in autofluorescence when the excitation wavelength was 400 or 405 nm. This discrepancy could be due to the lower excitation intensity of the xenon lamp, with its two monochromators, compared to lasers, which underscores the need to validate these observations with more accessible technologies, such as LEDs. The increased autofluorescence in i-RBCs could also be related to the conversion of the heme group to hemozoin by *P. falciparum*, suggesting that energy is dissipated through autofluorescence rather than being captured by the iron in hemoglobin, which normally acts as an electron acceptor. Other studies using two-photon excited autofluorescence (TPEF) have shown that hemoglobin exhibits emission in the Soret band (around 438 nm) with an extremely short half-life [30]. Sun et al., 2015, in a further study with TPEF of hemoglobin, confirmed that heme is the main fluorophore responsible for emission at ~430 nm, corresponding to the Soret band, and that it is independent of excitation wavelengths. Likewise, they showed that globin exhibited a maximum peak at ~350 nm. Furthermore, they stated that hemoglobin does not emit fluorescence under single-photon excitation since transitions are forbidden due to Laporte’s parity selection rule [31,32]. In contrast, our study demonstrated that single-photon excitation at 315 nm and 320 nm revealed autofluorescence peaks in the violet region (around 425 nm) and the blue region (around 450 nm), respectively. While these peaks are within the Soret band of the heme group, they could also be associated with other fluorophores present, and they are more pronounced in infected red blood cells (i-RBCs). The ability of hemoglobin to emit fluorescence through two-photon excitation, as demonstrated in the studies by W. Zheng et al. (2011) [30] and Sun et al. (2015) [31,32], suggests a promising approach for noninvasive imaging of erythrocytes through autofluorescence. However, for applications in malaria diagnosis, it is critical to validate the efficacy of autofluorescence detection at the wavelengths used in our study. This may open the possibility of developing fluorescence-based diagnostic methods using less complex and more accessible excitation equipment.

The shift in the Gaussian peaks observed in our results is indicative of changes in the chemical or structural environment of the fluorophores present in the cells due to infection. For example, in the UV region (from 338 to 388 nm), the Gaussian peaks show a red shift in both graphs in Figure 7a,b indicating that the infection has a consistent effect on the structure or composition of red blood cells.

The combination of spectroscopic techniques, such as autofluorescence, Raman spectroscopy [26], and IR spectroscopy [27,28], can significantly improve the sensitivity and accessibility of malaria diagnosis. The reviewed studies demonstrate that IR-based techniques have considerable potential for use in low-resource settings, while our autofluorescence findings provide a promising alternative. The integration of these technologies into portable diagnostic systems using UV and IR LEDs could revolutionize malaria detection, offering rapid and low-cost solutions for affected communities.

In terms of practical application, the identification of these specific wavelengths (315 and 320 nm) that effectively differentiate between *P. falciparum*-infected and uninfected red blood cells could facilitate the development of new diagnostic devices based on LED technology, which is more economical and accessible than lasers [35]. This is particularly relevant when compared to other advanced diagnostic methods, such as microfluidic systems [15,16,17], magnetophoresis and dielectrophoresis [52], microarrays [53], magneto-optical technology [54,55], and hematology analyzers such as the Sysmex XN-31 [19]. Although these methods are very accurate, they require expensive and complex equipment, limiting their applicability in low-resource regions where access to the health system is precarious.

The successful classification of infected samples in 91.67% of cases using Linear Discriminant Analysis highlights the potential of this approach to improve diagnostic rates compared to traditional methods, such as thick smear staining, which detects between 56% and 70% of cases [56], and up to 40% of infections identified via qPCR var ATS [13]. However, the exact mechanism that causes the increase in autofluorescence when i-RBCs are excited at 315 and 320 nm is not yet fully understood. It is possible that the breakdown of hemoglobin by the parasite generates additional fluorescent compounds by increasing the concentration of fluorophores (intrinsic fluorescent biomarkers) or that it alters the optical properties of the cell cytoplasm by increasing the emission efficiency of existing fluorophores in the infected cell, which would result in increased autofluorescence in the UV, violet, and blue regions.

The limitation observed in the differentiation of parasitemia levels reflects the need for further studies with more advanced excitation sources and larger numbers of samples representing different degrees of infection. Future research will seek to incorporate a wider spectral range and more powerful light sources to optimize the method.

## 5. Conclusions

In this study, we aimed to identify excitation wavelengths capable of differentiating normal red blood cells from *P. falciparum*-infected red blood cells using their autofluorescence, focusing on key wavelengths in the UV region. The results suggest that infected red blood cells exhibit significantly different autofluorescence behavior compared to uninfected cells when excited at 315 and 320 nm, showing increased UV peak intensity and shifts toward longer wavelengths. These variations could serve as biomarkers for detecting and studying infections, with the potential to distinguish *P. falciparum*-infected erythrocytes from healthy ones.

Compared to current methods, the findings support the feasibility of developing more affordable UV LED-based technologies with significant potential for diagnostic applications in low-resource settings. This preliminary study demonstrates the potential of autofluorescence for detecting *P. falciparum* infections. However, its capacity to distinguish different parasitemia levels should be assessed in future research. This will involve using higher-intensity light sources, validating findings with clinical samples, and optimizing the analyzed spectral range. Future developments should also account for individual variations in endogenous fluorophores. Priority research strategies include expanding sample sizes, normalizing signals, and identifying reliable biomarkers.

Finally, our results emphasize the need for continued research and development of diagnostic methods that are not only effective but also accessible and practical, encouraging their integration into portable devices for use in areas where malaria remains a major public health problem.

## Figures and Tables

**Table 1:** Summary of the characteristics of uninfected (u-RBC) and infected (i-RBC) red blood cell samples used in this study.

Sample Number	RBC	Medium	Strain of *P. falciparum*	Parasitemia (%)
1–6	Uninfected	EDTA	N/A	N/A
7	Infected	RPMI	FCR3	0.30
8	Infected	RPMI	FCR3	1.75
9	Infected	RPMI	FCR3	3.10
10	Infected	RPMI	FCR3	3.70
11	Infected	RPMI	FCR3	5.67
12	Infected	RPMI	FCB1	7.60

**Table 2:** Contribution of spectral variables to the first two principal components (PC1 and PC2) of the Principal Component Analysis (PCA). These ratios indicate that the two principal components adequately represent the significant differences between the signals.

	PC 1	PC 2
Exc_305	0.91694	−0.29296
Exc_310	0.30807	0.2695
Exc_315	0.17148	0.39064
Exc_320	0.13431	0.42394
Exc_325	0.093698	0.39272
Exc_330	0.065165	0.38752
Exc_335	0.034341	0.25178

**Table 3:** Characteristics of the spectral peaks for the two excitation wavelengths (λex). The parameters of the Gaussian peaks are shown, which include the central position of the peak (λc), the displacement of i-RBCs with respect to u-RBCs, the amplitude or intensity of each peak, the amplitude difference (u-RBC minus i-RBC), the percentage increase in fluorescence in i-RBCs, and the spectral region corresponding to the emission.

λ_ex_ (nm)	Peak	Sample	λ_c_ (nm)	Shift (nm)	Amplitude (RFU)	Difference (RFU)	Increase in i-RBCs (%)	Spectral Region
315	Gaussian 1	u-RBC	345.0	9	0.1738	0.1362	78%	UV
		i-RBC	354.0		0.3100			
	Gaussian 2	u-RBC	365.0	18	0.1500	−0.0100	−7%	UV
		i-RBC	383.0		0.1400			
	Gaussian 3	u-RBC	453.0	−3	0.0720	0.0230	32%	Blue
		i-RBC	450.0		0.0950			
	Gaussian 6	u-RBC	628.0	0	0.0375	0.0025	7%	Red
		i-RBC	628.0		0.0400			
320	Gaussian 1	u-RBC	353.6	1.4	0.1540	0.0660	43%	UV
		i-RBC	355.0		0.2200			
	Gaussian 2	u-RBC	370.0	8	0.0760	0.1780	234%	UV
		i-RBC	378.0		0.2540			
	Gaussian 3	u-RBC	425.0	−1	0.0760	0.0240	32%	Violet
		i-RBC	424.0		0.1000			
	Gaussian 4	u-RBC	468.0	7	0.0240	0.0310	129%	Blue
		i-RBC	475.0		0.0550			
	Gaussian 5	u-RBC	510.0	5	0.0200	0.0100	50%	Green
		i-RBC	515.0		0.0300			
	Gaussian 8	u-RBC	639.0	−0.1	0.0430	0.0000	0%	Red
		i-RBC	638.9		0.0430			

**Table 4:** Classification of the samples via Linear Discriminant Analysis, with a percentage of correctly classified cases of 91.67%.

Current Status	Group Size	Expected Status	
		i-RBC	u-RBC
i-RBC	12	10	2
u-RBC	12	0	12

**Figure A1:**
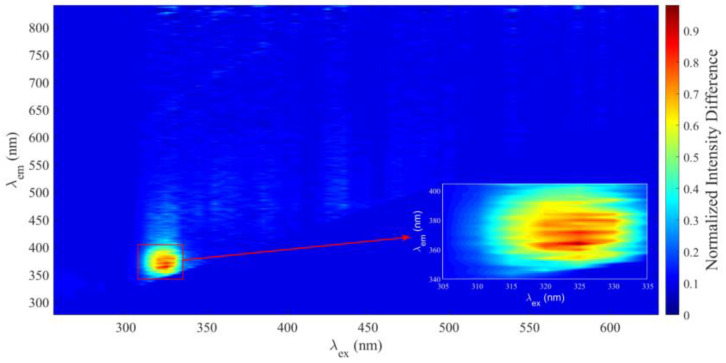
Normalized mean differences between i-RBC and u-RBC for strain 3D7.

**Figure A2:**
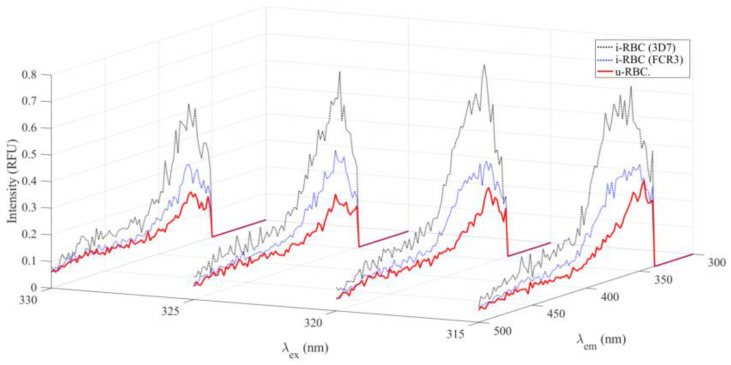
Fluorescence emission of u-RBCs and i-RBCs infected with *P. falciparum* strains FCR3 and 3D7.

**Figure A3:**
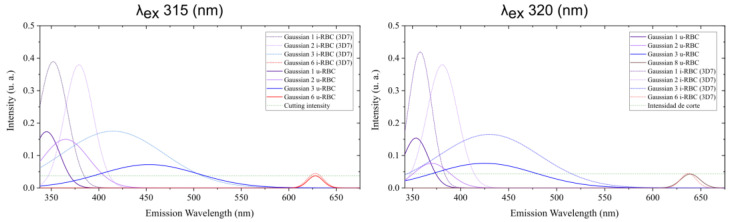
Autofluorescence peaks obtained by Gaussian fitting for the 3D7 strain.

## Data Availability

Inquiries can be directed to the corresponding author.

## Appendix A

### Appendix A.1. Complementary Analysis of P. falciparum Strain 3D7

#### Appendix A.1.1. Description of Samples

Six samples of uninfected red blood cells (u-RBC) and six samples infected (i-RBC) with the 3D7 strain of *Plasmodium falciparum*, provided by the Malaria Group of UdeA, were analyzed. These samples were prepared following the same protocol described in Section 2 of “Materials and Methods” for the FCR3 strain.

#### Appendix A.1.2. Main Results

Normalized differences: The average normalized differences between i-RBC and u-RBC for the 3D7 strain were within a comparable range to those observed for the FCR3 strain, with the highest values occurring at the 315 and 320 nm wavelengths (see Figure A1).

Spectral analysis: The results indicated that similar to the FCR3 strain, samples infected with the 3D7 strain exhibited increased autofluorescence in the UV, violet, and blue regions compared to uninfected samples. Moreover, the 3D7 strain demonstrated even higher fluorescence levels than the FCR3 strain (see Figure A2).

Peak distribution: Significant autofluorescence peaks were observed in the same spectral ranges as those in the FCR3 strain, reinforcing the consistency of the results (see Figure A3).

#### Appendix A.1.3. Statistical Validation

PCA, LDA, LOOCV, and k-Fold analyses included combined data from both strains (12 healthy and 12 infected samples). This increased the variability represented and allowed an average accuracy of 91.7% in LOOCV and 92% ± 9.8% in k-Fold.

## References

[1] Ramasamy R. (2014). Zoonotic malaria—Global overview and research and policy needs. Front. Public Health.

[2] (2023). WHO World Malaria Report 2023 Geneva: World Health Organization. https://www.who.int/teams/global-malaria-programme/reports/world-malaria-report-2023.

[3] Amexo M., Tolhurst R., Barnish G., Bates I. (2004). Malaria misdiagnosis: Effects on the poor and vulnerable. Lancet.

[4] Tangpukdee N., Duangdee C., Wilairatana P., Krudsood S. (2009). Malaria diagnosis: A brief review. Korean J. Parasitol..

[5] Oyeyemi O.T., Ogunlade A.F., Oyewole I.O. (2015). Comparative assessment of microscopy and rapid diagnostic test (RDT) as malaria diagnostic tools. Res. J. Parasitol..

[6] Madkhali A.M., Ghzwani A.H., Al-Mekhlafi H.M. (2022). Comparison of Rapid Diagnostic Test, Microscopy, and Polymerase Chain Reaction for the Detection of *Plasmodium falciparum* Malaria in a Low-Transmission Area, Jazan Region, Southwestern Saudi Arabia. Diagnostics.

[7] Berzosa P., de Lucio A., Romay-Barja M., Herrador Z., González V., García L., Fernández-Martínez A., Santana-Morales M., Ncogo P., Valladares B. (2018). Comparison of three diagnostic methods (microscopy, RDT, and PCR) for the detection of malaria parasites in representative samples from Equatorial Guinea. Malar. J..

[8] Shankar H., Singh M.P., Phookan S., Singh K., Mishra N. (2021). Diagnostic performance of rapid diagnostic test, light microscopy and polymerase chain reaction during mass survey conducted in low and high malaria-endemic areas from two North-Eastern states of India. Parasitol. Res..

[9] Charpentier E., Benichou E., Pagès A., Chauvin P., Fillaux J., Valentin A., Guegan H., Guemas E., Salabert A.S., Armengol C. (2020). Performance evaluation of different strategies based on microscopy techniques, rapid diagnostic test and molecular loop-mediated isothermal amplification assay for the diagnosis of imported malaria. Clin. Microbiol. Infect..

[10] Feleke D.G., Alemu Y., Yemanebirhane N. (2021). Performance of rapid diagnostic tests, microscopy, loop-mediated isothermal amplification (LAMP) and PCR for malaria diagnosis in Ethiopia: A systematic review and meta-analysis. Malar. J..

[11] Slater H.C., Ding X.C., Knudson S., Bridges D.J., Moonga H., Saad N.J., De Smet M., Bennett A., Dittrich S., Slutsker L. (2022). Performance and utility of more highly sensitive malaria rapid diagnostic tests. BMC Infect. Dis..

[12] Yigezu E., Wondale B., Abebe D., Tamiru G., Eligo N., Lindtjørn B., Gadisa E., Tadesse F.G., Massebo F. (2023). Malaria misdiagnosis in the routine health system in Arba Minch area district in southwest Ethiopia: An implication for malaria control and elimination. Malar. J..

[13] Opoku Afriyie S., Addison T.K., Gebre Y., Mutala A.H., Antwi K.B., Abbas D.A., Addo K.A., Tweneboah A., Ayisi-Boateng N.K., Koepfli C. (2023). Accuracy of diagnosis among clinical malaria patients: Comparing microscopy, RDT and a highly sensitive quantitative PCR looking at the implications for submicroscopic infections. Malar. J..

[14] Zheng Z., Cheng Z. (2017). Advances in Molecular Diagnosis of Malaria. Adv. Clin. Chem..

[15] Wu W.T., Martin A.B., Gandini A., Aubry N., Massoudi M., Antaki J.F. (2016). Design of microfluidic channels for magnetic separation of malaria-infected red blood cells. Microfluid. Nanofluid..

[16] Warkiani M.E., Tay A.K.P., Khoo B.L., Xiaofeng X., Han J., Lim C.T. (2014). Malaria detection using inertial microfluidics. Lab Chip.

[17] Li J., Saidi A.M., Seydel K., Lillehoj P.B. (2024). Rapid diagnosis and prognosis of malaria infection using a microfluidic point-of-care immunoassay. Biosens. Bioelectron..

[18] Campuzano-Zuluaga G., Hänscheid T., Grobusch M.P. (2010). Automated haematology analysis to diagnose malaria. Malar. J..

[19] Zuluaga-Idárraga L., Rios A., Sierra-Cifuentes V., Garzón E., Tobón-Castaño A., Takehara I., Toya Y., Izuka M., Uchihashi K., Lopera-Mesa T.M. (2021). Performance of the hematology analyzer XN-31 prototype in the detection of *Plasmodium* infections in an endemic region of Colombia. Sci. Rep..

[20] Kagaya W., Takehara I., Kurihara K., Maina M., Chan C.W., Okomo G., Kongere J., Gitaka J., Kaneko A. (2022). Potential application of the haematology analyser XN-31 prototype for field malaria surveillance in Kenya. Malar. J..

[21] Wongsrichanalai C., Barcus M.J., Muth S., Sutamihardja A., Wernsdorfer W.H. (2007). A Review of Malaria Diagnostic Tools: Microscopy and Rapid Diagnostic Test. Am. J. Trop. Med. Hyg..

[22] Dumoulin P.C., Trop S.A., Ma J., Zhang H., Sherman M.A., Levitskaya J. (2015). Flow cytometry-based detection and isolation of *Plasmodium falciparum* liver stages in vitro. PLoS ONE.

[23] Yamada K., Yamamoto T., Sasaki K., Huber A.R., Brunner-Agten S. (2017). Feasibility of Measuring Autofluorescence of Red Blood Cells Utilizing a Novel Flow Cytometer to Define Iron Deficiency Patients. Sysmex J. Int..

[24] Campo J.J., Aponte J.J., Nhabomba A.J., Sacarla J., Angulo-Barturen I., Jiménez-Díaz M.B., Alonso P.L., Dobano C. (2011). Feasibility of flow cytometry for measurements of *Plasmodium falciparum* parasite burden in studies in areas of malaria endemicity by use of bidimensional assessment of YOYO-1 and autofluorescence. J. Clin. Microbiol..

[25] Cai C., Carey K.A., Nedosekin D.A., Menyaev Y.A., Sarimollaoglu M., Galanzha E.I., Stumhofer J.S., Zharov V.P. (2016). In vivo photoacoustic flow cytometry for early malaria diagnosis. Cytom. Part A.

[26] Goh B., Ching K., Soares Magalhães R.J., Ciocchetta S., Edstein M.D., Maciel-de-freitas R., Sikulu-Lord M.T. (2021). The application of spectroscopy techniques for diagnosis of malaria parasites and arboviruses and surveillance of mosquito vectors: A systematic review and critical appraisal of evidence. PLoS Negl. Trop. Dis..

[27] Khoshmanesh A., Dixon M.W.A., Kenny S., Tilley L., Mcnaughton D., Wood B.R. (2014). Detection and Quantification of Early-Stage Malaria Parasites in Laboratory Infected Erythrocytes by Attenuated Total Reflectance Infrared Spectroscopy and Multivariate Analysis. Anal. Chem..

[28] Mshani I.H., Siria D.J., Mwanga E.P., Sow B.B.D., Sanou R., Opiyo M., Sikulu-Lord M.T., Ferguson H.M., Diabate A., Wynne K. (2023). Key considerations, target product profiles, and research gaps in the application of infrared spectroscopy and artificial intelligence for malaria surveillance and diagnosis. Malar. J..

[29] Shrirao A.B., Schloss R.S., Fritz Z., Shrirao M.V., Rosen R., Yarmush M.L. (2021). Autofluorescence of blood and its application in biomedical and clinical research. Biotechnol. Bioeng..

[30] Zheng W., Li D., Zeng Y., Luo Y., Qu J.Y. (2011). Two-photon excited hemoglobin fluorescence. Biomed. Opt. Express..

[31] Sun Q., Zeng Y., Zhang W., Zheng W., Luo Y., Qu J.Y. (2015). Two-photon excited fluorescence emission from hemoglobin. Multiphot. Microsc. Biomed. Sci. XV.

[32] Sun Q., Zheng W., Wang J., Luo Y., Qu J.Y. (2015). Mechanism of two-photon excited hemoglobin fluorescence emission. J. Biomed. Opt..

[33] Bukara K., Jovanić S.Z., Drvenica I.T., Stančić A., Ilić V., Rabasović M.D., Pantelić D., Jelenković B., Bugarski B., Krmpot A.J. (2017). Mapping of hemoglobin in erythrocytes and erythrocyte ghosts using two photon excitation fluorescence microscopy. J. Biomed. Opt..

[34] Peng C., Liu J. (2013). Studies on Red-Shift Rules in Fluorescence Spectra of Human Blood Induced by LED. Appl. Phys. Res..

[35] Opoku-Ansah J., Eghan M.J., Anderson B., Boampong J.N. (2014). Wavelength Markers for Malaria (*Plasmodium falciparum*) Infected and Uninfected Red Blood Cells for Ring and Trophozoite Stages. Appl. Phys. Res..

[36] Serebrennikova Y.M., Patel J., Milhous W.K., García-Rubio L.H. (2010). Quantitative analysis of morphological alterations in *Plasmodium falciparum* infected red blood cells through theoretical interpretation of spectral measurements. J. Theor. Biol..

[37] Opoku-Ansah J., Eghan M.J., Anderson B., Boampong J.N., Buah-Bassuah P.K. (2016). Laser-Induced Autofluorescence Technique for *Plasmodium falciparum* Parasite Density Estimation. Appl. Phys. Res..

[38] Garrido-Tamayo M.Á., Pedraja-Rejas L., Tiutiunnyk Y., Hoyos F.E., Laroze D. (2024). Mapping the research landscape in the diagnosis of *Plasmodium falciparum* malaria: Insights from a bibliometric study. Healthcare.

[39] WHO Giemsa Staining of Malaria Blood Films (2016). Malaria Microscopy Standard Operating Procedure—MM-SOP-07A. http://www.wpro.who.int/mvp/lab_quality/2096_oms_gmp_sop_07a_rev.pdf.

[40] WHO (2016). Malaria Parasite Counting Malaria Microscopy Standard Operating Procedure—MM-SOP-09. https://www.who.int/publications/i/item/HTM-GMP-MM-SOP-09.

[41] Vernot-Hernandez J.P., Heidrich H.G. (1985). The relationship to knobs of the 92,000 D protein specific for knobby strains of *Plasmodium falciparum*. Z. Parasitenkd..

[42] Hammer Ø., Harper D.A.T., Ryan P.D. (2001). PAST: Paleontological Statistics Software Package for Education and Data Analysis. Paleaontología Electrónica.

[43] Hobro A.J., Pavillon N., Fujita K., Ozkan M., Coban C., Smith N.I. (2015). Label-free Raman imaging of the macrophage response to the malaria pigment hemozoin. Analyst.

[44] Molyneux P.M., Kilvington S., Wakefield M.J., Prydal J.I., Bannister N.P. (2015). Autofluorescence Signatures of Seven Pathogens: Preliminary in Vitro Investigations of a Potential Diagnostic for Acanthamoeba Keratitis. Cornea.

[45] Laura-Ochoa L. Evaluación de Algoritmos de Clasificación utilizando Validación Cruzada. Proceedings of the 17th LACCEI International Multi-Conference for Engineering, Education, and Technology.

[46] Silva A., Godínez J., Fernández M., Haro E. (2009). Espectroscopía de fluorescencia inducida por láser en células. La Física Biológica en México: Temas Selectos.

[47] Dhamnetiya D., Goel M.K., Jha R.P., Shalini S., Bhattacharyya K. (2022). How to Perform Discriminant Analysis in Medical Research? Explained with Illustrations. J. Lab. Physicians.

[48] Appolus E.E., Okoli C.N. (2022). A Robust Comparison Powers of Four Multivariate Analysis of Variance Tests. Eur. J. Stat. Probab..

[49] Preißinger K., Molnar P., Vertessy B., Kezsmarki I., Kellermayer M. (2021). Stage-Dependent Topographical and Optical Properties of *Plasmodium falciparum*-Infected Red Blood Cells. J. Biotechnol. Biomed..

[50] Masilamani V., Devanesan S., Ravikumar M., Perinbam K., AlSalhi M., Prasad S., Palled S., Ganesh K.M., Alsaeed A.H. (2014). Fluorescence spectral diagnosis of malaria a preliminary study. Diagn. Pathol..

[51] Bellemare M.J., Bohle D.S., Brosseau C.N., Georges E., Godbout M., Kelly J., Leimanis M.L., Leonelli R., Olivier M., Smilkstein M. (2009). Autofluorescence of condensed heme aggregates in malaria pigment and its synthetic equivalent hematin anhydride (B-hematin). J. Phys. Chem. B.

[52] Kasetsirikul S., Buranapong J., Srituravanich W., Kaewthamasorn M., Pimpin A. (2016). The development of malaria diagnostic techniques: A review of the approaches with focus on dielectrophoretic and magnetophoretic methods. Malar. J..

[53] Hashimoto M., Yatsushiro S., Yamamura S., Kataoka M. (2017). Development of a cell microarray chip system for early and accurate malaria diagnosis. Synth. Engl. Ed..

[54] Butykai A., Orbán A., Kocsis V., Szaller D., Bordács S., Tátrai-Szekeres E., Kiss L.F., Bóta A., Vértessy B.G., Zelles T. (2013). Malaria pigment crystals as magnetic micro-rotors: Key for high-sensitivity diagnosis. Sci. Rep..

[55] Mens P.F., Matelon R.J., Nour B.Y.M., Newman D.M., Schallig H.D.F.H. (2010). Laboratory evaluation on the sensitivity and specificity of a novel and rapid detection method for malaria diagnosis based on magneto-optical technology (MOT). Malar. J..

[56] Lema O.E., Carter J.Y., Nagelkerke N., Wangai M.W., Kitenge P., Gikunda S.M., Arube P.A., Munafu C.G., Materu S.F., Adhiambo C.A. (1999). Comparison of five methods of malaria detection in the outpatient setting. Am. J. Trop. Med. Hyg..

